# Geographic distribution of C_4_ species and its phylogenetic structure across China

**DOI:** 10.3389/fpls.2023.1214980

**Published:** 2023-06-08

**Authors:** Aiying Zhang, Zhongjie Yang, Yu Zuo, Liang Ma, Hanyu Zhang

**Affiliations:** ^1^College of Life Sciences, China Jiliang University, Hangzhou, China; ^2^Mineral Resources and Geological Survey Center, Shaanxi Institute of Geological Survey, Xi’an, China

**Keywords:** clustering, niche conservatism, phylogenetic distance, phylogenetic diversity, Poaceae, standardized effect size

## Abstract

Over the past fifty years, the distribution patterns of C_4_ species, across large spatial scales, are largely ignored. Here, we endeavored to examine patterns in the taxonomic and phylogenetic diversity of species with C_4_ photosynthetic pathways across the broad spatial extent of China and relate those to climatic gradients. We built a database of all plants with the C_4_ photosynthetic pathway in China. We analyzed the geographic distributions, taxonomic diversity, phylogenetic diversity, and phylogenetic structure of all C_4_ species, as well as the three families with the most C_4_ species (Poaceae, Amaranthaceae and Cyperaceae), and compared their values along temperature and precipitation gradients at two scales—the level of the province and at the 100 x 100 km grid cell. We found 644 C_4_ plants (belonging to 23 families 165 genera) in China, with Poaceae (57%), Amaranthaceae (17%), Cyperaceae (13%) accounting for the majority of species. Standardized effect size values of phylogenetic distances were negative overall, indicating that C_4_ species showed a phylogenetic clustering pattern. Southern China had the highest species richness and the highest degree of phylogenetic clustering. C_4_ tended to be more phylogenetically over-dispersed in regions with colder and/or drier climates, but more clustered in warmer and/or wetter climates. Patterns within individual families were more nuanced. The distribution of C_4_ species and its phylogenetic structure across China was constrained by temperature and precipitation. C_4_ species showed a phylogenetic clustering pattern across China, while different families showed more nuanced responses to climate variation, suggesting a role for evolutionary history.

## 1 Introduction

Although they represent a small percentage of vascular plant species on the earth (~0.005%) (Sieben et al., 2010), C_4_ plants have highly efficient photosynthesis and can play an important role in many ecosystems, such as during succession and recovery of degraded ecosystems (Wang, 2002a; Wang, 2002b). Moreover, the occurrences of C_4_ plants are good indicators of ecosystem variation because they tend to be more tolerant of stressful environments than C_3_ plants (Wang and Ma, 2016; Pardo and VanBuren, 2021; Zhang and Xie, 2021). Plants with C_4_ photosynthesis not only differ biochemically from species with other photosynthetic systems, but also in their evolutionary history and ecological interactions (Osborne et al., 2015; Sage, 2017). Therefore, exploring the patterns in the distribution of C_4_ plants and their phylogenetic relationships across large spatial scales provides basic biogeographic understanding of this important plant trait both ecologically and evolutionarily.

The geographic distribution of C_4_ plants also tends to be associated with different environmental conditions (Munson and Long, 2017). For example, C_4_ plants tend to be more prevalent and diverse in warmer tropical areas and are notably absent from cooler regions (Osborne et al., 2015). However, to date, most studies on the distribution and diversity of C_4_ plants have tended to focus only on relatively small geographic areas (Taylor et al., 2010), or have examined it only within a subset of the angiosperm phylogeny (e.g., in grasses) (Edwards and Still, 2008; Visser et al., 2012; Sonawane et al., 2021), while an exploration of patterns of species diversity, phylogenetic diversity and phylogenetic structure could provide insights into the interactions between ecological and evolutionary processes influencing the distribution and diversity of these species (Liu et al., 2012; Munroe et al., 2022).

By examining patterns of species richness of C_4_ plants, together with patterns of phylogenetic diversity (which incorporates evolutionary relationships) and patterns of phylogenetic structure (i.e., clustering of co-occurring species within the phylogeny), we can make progress in understanding the patterns, and potential processes influencing those patterns. For example, because C_4_ plants tend to have evolved in warmer, more tropical regions (Osborne et al., 2015), the tropical niche conservatism hypothesis (i.e., freezing-tolerance hypothesis) (Latham and Ricklefs, 1993; Wiens and Donoghue, 2004) may help to describe patterns of species richness if there is a strong latitudinal gradient in C_4_ species. Likewise, the phylogenetic structure of species assemblages, such as how clustered co-occurring species are within the phylogeny, can reflect their evolutionary history of adaptation and tolerance to environments (Qian et al., 2019). For example, the phylogenetic niche conservatism hypothesis predicts that closely related species have more similar niches and thus should share similar environmental conditions and geographic distributions (Pyron et al., 2015; Lu et al., 2018; Qian et al., 2022).

Here, we endeavored to more deeply understand the diversity, structure and distribution of species of species with C_4_ photosynthesis across broad geographic areas and among the entire angiosperm phylogeny, as well as within specific groups within angiosperms that had large numbers of C_4_ species. To do so, we took advantage of an extensive database of C_4_ species and their distributions from across China. China provides an important venue in which to examine patterns of diversity and structure within C_4_ species, because it is a geographically and climatically diverse country, ranging from cold temperate to tropical ecosystems, and from extremely wet rainforests to deserts, with a well-characterized flora. Using these data, we could examine patterns of the taxonomic (species) richness of C_4_ taxa and its relation to climatic variables. In addition, by combining species distribution and composition data with information on species phylogenies, we could examine patterns of phylogenetic diversity of species, as well as the phylogenetic structure (e.g., clustering) of co-occurring species within the phylogeny. Overall, we found that the distribution of C_4_ species richness, phylogenetic diversity phylogenetic structure across China was constrained by temperature and precipitation; C_4_ species showed a phylogenetic clustering pattern across China, while different families showed more nuanced responses to climate variation, suggesting a role for evolutionary history.

## 2 Materials and methods

### 2.1 Data compilation

We compiled a database of C_4_ plants in China from a number of published references (Supporting Information, **Table S1**). Specifically, we classified C_4_ species as such when their photosynthetic pathway was identified by stable carbon isotope ratios (10‰-15‰, Sage and Monson, 1999) and/or anatomy (Kranz anatomy, Osborne et al., 2015). When a species photosynthetic pathway was not known from stable carbon isotopes or anatomical type, we classified these unknown species as C_4_ species if we could find such information in other databases, and by assuming that all species within a genus should share the same photosynthetic pathway (Osborne et al., 2015) (though we recognize that this is a coarse approach and might misclassify some species). We standardized species names from published references according to the Flora of China (http://foc.iplant.cn/) and the World Flora Online (www.world.flora.online.org). We classified species into family based on the latest Angiosperm Phylogeny Group classification (The Angiosperm Phylogeny Group, 2016).

We obtained species distribution data within regions from the Flora of China (**Table S1**). Furthermore, we obtained occurrence records with coordinates (latitude and longitude) from multiple sources, including the Chinese Virtual Herbarium (https://www.cvh.ac.cn/), National Specimen Information Infrastructure (http://www.nsii.org.cn/2017/home.php) and the Global Biodiversity Information Facility (https://www.gbif.org/). We then integrated these species distribution data so that we could estimate patterns of diversity and co-occurrence of species at two scales—the province level and at the level of 100 km x 100 km grid cells. For the province level, we divided China into 29 provinces, by combing Beijing and Tianjin within the Hebei Province, Hong Kong and Macau within the Guangdong province, and Shanghai within the Jiangsu Province.

In order to compare patterns of C_4_ diversity and phylogenetic structure with climate variables, we extracted climate data from the WorldClim database (http://www.worldclim.com/version2). Specifically, we extracted annual mean temperature (hereafter temperature) and annual mean precipitation (hereafter precipitation), which are known to be the most important bioclimatic variables driving plant species distributions in China (Qian et al., 2022). We used ArcGIS 10.8 to extract the variables from each province and grid cell.

### 2.2 Indices of species richness and phylogenetic structure

For each index of diversity or structure, we calculated the value for the entire group of C_4_ species, as well as separately for C_4_ species in the three families that accounted for 87% of all C_4_ species—Poaceae, Amaranthaceae and Cyperaceae (**Figure S1**). We calculated species richness as the total number of species that co-occur within a given province or grid cell. In order to calculate patterns of phylogenetic diversity and structure, we first built a phylogenetic tree of all C_4_ species in our database, using the R package *‘V.PhyloMaker2’* (Jin and Qian, 2022). With this phylogeny in hand, we calculated three indices of phylogenetic structure. First, we calculated Faith’s phylogenetic diversity (PD), which calculates the sum of total phylogenetic branch lengths of species in an assemblage (Faith, 1992). Second, we calculated the mean pairwise phylogenetic distances (MPD) between all possible pairs of species in an assemblage, which quantifies overall clustering of taxa on a tree (Webb et al., 2002). Third, we calculated the mean phylogenetic distance to the nearest taxon (MNTD) for each taxon in an assemblage, which quantifies the extent of terminal clustering, independent of deep level clustering (Webb et al., 2002; Qian et al., 2019). For each phylogenetic index, we calculated its standardized effect size (SES) to quantify the degree to which phylogenetic diversity and phylogenetic relatedness between species differed from what would have been expected if species were randomly distributed across the geographical extent. The SES was calculated as X = (X_observed_-meanX_randomized_)/(sdX_randomized_), where X_observed_ represents the observed PD, MPD and MNTD values in a species assemblage, while meanX_randomized_ and sdX_randomized_ represent the expected mean and standard deviation of the species from the randomized assemblages (Webb et al., 2002). We ran each null randomization 999 times with a fixed species richness. Higher values of the standardized effect size of phylogenetic diversity (SES.PD) indicate a higher phylogenetic diversity (Qian et al., 2019). For the standardized effect size of the mean phylogenetic distance (SES.MPD), negative values indicate that the observed MPD values were lower than randomized ones and that species are more closely related than expected from the entire species pool (i.e., species are phylogenetically clustered). Conversely, positive SES.MPD values indicate that species are phylogenetically over-dispersed. Likewise, negative values of the standardized effect size of the mean phylogenetic distance to the nearest taxon (SES.MNTD) indicate clustering within the terminal part of the phylogenetic tree, while positive values indicate overdispersion. All phylogenetic indices and their SES were calculated using the R package *‘picante’* (Kembel et al., 2010).

### 2.3 Statistical analysis

At both the level of the province and the grid level, we used Spearman’s rank correlation analysis to test the relationship between species richness, the indices of phylogenetic structure, and the two bioclimatic variables (temperature and precipitation). We also calculated phylogenetic differences (MPD and MNTD) between regions using the R package *‘picante’* with the function *comdist* and *comdistnt* (Kembel et al., 2010), which we visualized using Principal Coordinate Analysis (PCoA).

All statistical analyses and figure construction were conducted in R 4.2.0 (R Core Team, 2022).

## 3 Results

### 3.1 Overview of the C_4_ dataset

Overall, we identified 644 C_4_ vascular plant species (belonging to 23 families 165 genera, **Table S1**; **Figure S1**) distributed in China, including 371 where species were directly measured to be C_4_, and 273 of which we inferred to be C_4_ based on the literature or their congeners. The Poaceae accounted for more than half of all C_4_ species (57%), with 17% of C_4_ species in the Amaranthaceae and 13% in the Cyperaceae in the third (13%); the remaining 13% of species were spread among 20 other families. Most C_4_ plants were perennial (50%) and annual (42%) non-woody (herbaceous) species, while 9% of species were shrubs (**Figure S2A**). Finally, there were 65 (~10%) C_4_ species that were non-native to China, half of which were classified as invasive species (**Figure S2B**).

**Figure 1:**
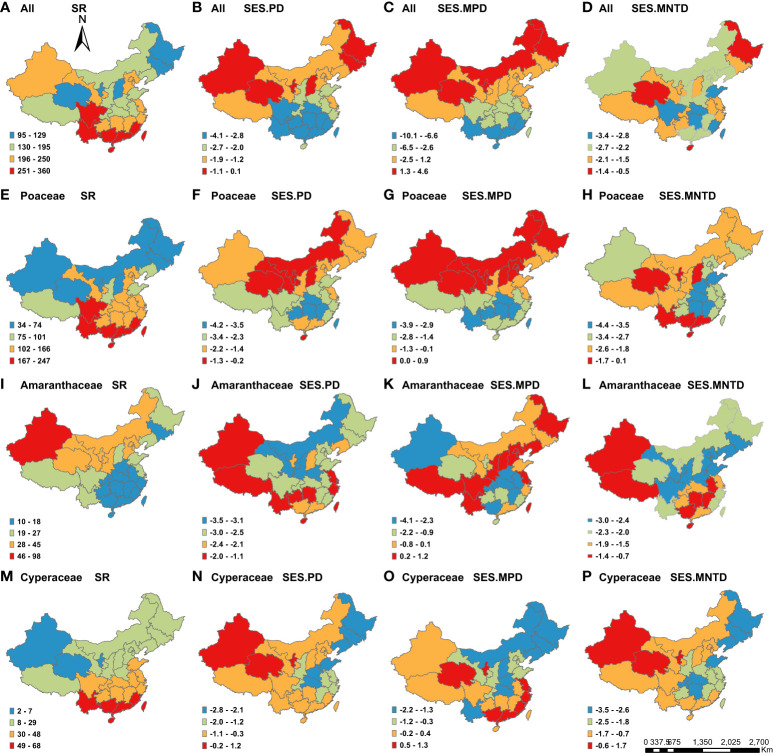
Geographic patterns of species richness (SR) and the standardized effect size values of phylogenetic structure indices (SES.PD, SES.MPD and SES.MNTD) for C_4_ plants across the 29 Chinese provinces. Panels represent different metrics (columns) and different taxonomic groupings (all C_4_ species, Poaceae, Amaranthaceae and Cyperaceae). The map of China is incomplete, which only includes mainland, Taiwan Island and Hainan Island.

The distribution of C_4_ species was skewed across China, with most species occurring in the south: Yunnan and Guangdong provinces had more than 50% of all C_4_ species, while 40% were in Guangxi, Fujian, Taiwan, Hainan and Sichuan (**Figure 1**). There were 99 C_4_ species that were endemic to only one province, more than half of which were in Xinjiang (61%) (including 35 species in the Amaranthaceae) (**Figure S3**).

### 3.2 Distribution patterns of species richness and phylogenetic structure

Overall, we found that both species richness and PD varied greatly across China for all species, as well as the three most common families, across the 29 provinces, while MPD and MNTD varied less (**Table S2**). Among the different families, the C_4_ species in the Amaranthacea had the highest Coefficient of Variation (CV) for species richness (71.4%), PD (55.4%) and MPD (19.9%) among the provinces. The C_4_ species in the Cyperaceae had the highest CV for MNTD (74.3%).

We found that species richness was highest in the south for all C_4_ species (**Figure 1A**), as well as those in the Poaceae (**Figure 1E**) and Cyperaceae (**Figure 1M**). Meanwhile, the highest richness of C_4_ species in the Amaranthaceae species was in the northwestern part of China (**Figure 1I**). The regions with the highest PD and SES.PD for all C_4_ species (**Figure 1B**), as well as those in the Poaceae (**Figure 1F**), were in northern China. For C_4_ species in the Amaranthaceae, the highest SES.PD was located in the northwestern, southwestern and southeastern China (**Figure 1J**), while northwestern China had the highest SES.PD for C_4_ species in the Cyperaceae (**Figure 1N**).

For patterns of phylogenetic structure, we found that northeastern China had the highest positive SES.MPD and SES.MNTD (**Figures 1C, G, K**), as well as the lowest negative SES.MPD and SES.MNTD (**Figures 1L, O, P**), indicating that this region had the highest phylogenetic overdispersion. Meanwhile, northwestern China had the highest positive SES.MPD and SES.MNTD (**Figures 1C, G**), as well as the highest negative SES.MPD and SES.MNTD), consistent with the highest phylogenetic overdispersion (and lowest clustering). Southern China had the lowest negative SES.MPD and SES.MNTD (**Figures 1C, D, G, K**), indicated that it had the highest phylogenetic clustering.

**Figure 2:**
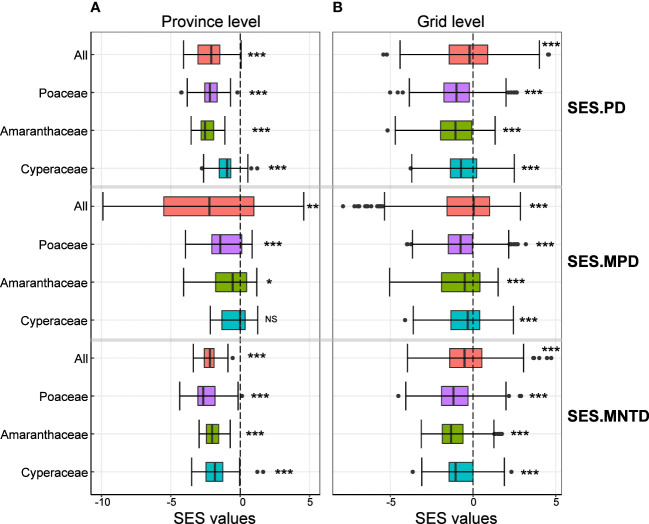
Variation in the standardized effect size values of phylogenetic structure indices at the province level (left column) and grid level (right colum). Values of significant differences from zero are shown by three asterisks (*P* < 0.001), two asterisks (0.001< *P* < 0.01), one asterisk (0.01< *P* < 0.05) and NS (*P* > 0.05).

We found that the mean values of SES.PD, SES.MPD and SES.MNTD of all C_4_ species were significantly less than zero at both the province and grid level (**Figures 2A, B**), indicating that C_4_ plants were phylogenetically clustered. The same was true when we analyzed each of the most common families separately, except for the SES.MPD of the C_4_ species in the Cyperaceae at the province level (**Figures 2A, B**).

### 3.3 Relationships between species richness, phylogenetic structure and bioclimatic variables

**Figure 3:**
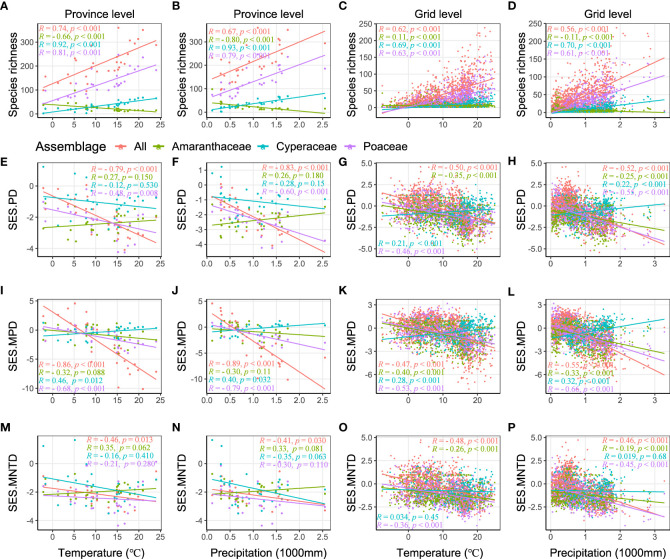
Relationships of species richness and SES values of phylogenetic structure indices (SES.PD, SES.MPD, and SES.MNTD) with temperature and precipitation, calculated by Spearman’s rank correlation. *R* is correlation coefficient, *p* indicates significance.

We found significant positive relationships between the all C_4_ species richness, as well as both the Poaceae and Cyperacae, and both temperature and precipitation at the province and grid level (**Figures 3A–D**). The species richness of C_4_ species in the Amaranthaceae had a negative relationship with precipitation at both the province and grid level, but with non-consistent relationships with temperate at the province level and grid level (**Figures 3A–D**). On the other hand, the SES.PD of all C_4_ species and the C_4_ species in the Poaceae had a significant negative relationship with temperature and precipitation at both the province and grid level (**Figures 3E–H**), as did the SES.PD of the C_4_ species in the Amaranthaceae had a significant negative relationship at the grid level (but not province level) and the C_4_ species in the Cyperaceae had a significant positive relationship at the grid level (but not province level) (**Figures 3E–H**). Patterns of SES.MPD were similar to those of SES.PD (**Figures 3I–L**). For SES.MNTD, we found that all C_4_ species decreased a significant negative relationship with temperature and precipitation at both the province and grid level and the C_4_ species of the Cyperaceae had no significant relationship with temperature and precipitation (**Figures 3M–P**).

**Figure 4:**
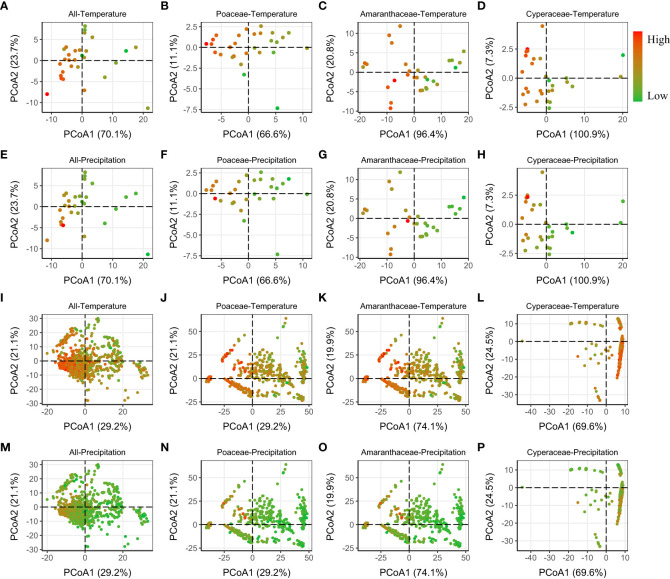
Relative phylogenetic differences (MNTD) among the regions [province level **(A–H)** and grid level **(I–P)**], visualized using a PCoA ordination biplot, calculated using the Gower distance. The color of dots varied from green to red, meaning the Temperature/Precipitation increased from low to high. Similar results of MPD (whose first two axes explained much lower variance than MNTD) were showed in **Figure S4**.

Finally, we found that the PCoA showed that phylogenetic distance (MNTD and MPD) between regions with high temperature (and/or lower precipitation) were smaller, and thus more phylogenetically similar (**Figures 4A–P**; **S4–S6**).

## 4 Discussion

Overall, our study showed that there is considerable, but important, variation in the distribution, diversity and phylogenetic structure of plants with C_4_ photosynthesis across the broad geographical (and climatic) extent of China. More specifically, we found that C_4_ species accounted for about 2% of all angiosperms in China (644 C_4_ species out of 29716-30260 total angiosperm species, Wang et al., 2015; Qian et al., 2022). Furthermore, the C_4_ photosynthetic pathway was most prevalent among species in the Poaceae. About half of all Poaceae species are C_4_ (Osborne et al., 2015), and our study, like others (Sage and Monson, 1999), found that Poaceae with C_4_ photosynthesis accounted for about half of all C_4_ species.

Our main result was that C_4_ species had higher species richness in regions with warmer and/or wetter climates. This pattern also holds when we examined patterns of the C_4_ species within the Poaceae and Cyperaceae. This is consistent with ideas related to the tropical niche conservatism hypothesis (Wiens and Donoghue, 2004) and is also mirrored by patterns in the distribution of woody and seed plants in China (Wang et al., 2011; Qian et al., 2019). C_4_ species in the Amaranthaceae, however, had a higher species richness in northwestern China in regions with drier climates (see also Wang and Ma, 2016; Rudov et al., 2020; Munroe et al., 2022). This suggests that the evolutionary history of C_4_ species within the Amaranthaceae likely differs from the other families with C_4_ photosynthesis.

While we found that C_4_ species in the Cyperaceae were more phylogenetically clustered in regions with colder and/or drier climates, consistent with previous studies in China (Lu et al., 2018; Qian et al., 2019) and North America (Qian and Sandel, 2017), this pattern did not hold for the other groups. Instead, we found that all C_4_ species (as well as those within the Poaceae and Amaranthaceae) tended to be more phylogenetically over-dispersed in regions with colder and/or drier climates, but more clustered in warmer and/or wetter climates.

These results are also consistent with the phylogenetic niche conservatism hypothesis (Qian et al., 2019), where all C_4_ species in the warmer and wetter climates of southern China had the highest species richness, but relatively lower phylogenetic diversity and the higher phylogenetic clustering, suggesting that species here were highly closely related. However, patterns differed for the C_4_ species in the Amaranthaceae, where drier climates of the northwestern parts of China had the highest species richness and phylogenetic diversity, as well as low phylogenetic clustering, suggesting these species were relatively distantly related. On the other hand, C_4_ species in the Cyperaceae had lower species richness, highest phylogenetic diversity and high phylogenetic overdispersion in the colder climates of the northwest, indicating these species were relatively distantly related. Overall, this suggests that C_4_ species in different families respond different to geographic gradients, likely as a result of their different evolutionary histories (Munroe et al., 2022).

We should emphasize, however, that while ours is the most comprehensive analysis of the distribution and diversity of C_4_ species in China, there are still some improvements that can be made. For example, in our database, there are 273 species whose stable carbon isotope value or anatomical type were not given, but rather inferred based on other databases and the assumption that all species within one genus should share the same photosynthetic pathway (Osborne et al., 2015). However, it is possible that taxonomic changes could influence this assumption. For instance, species in the *Potamogeton* genus were identified with C_4_ photosynthetic pathway, except for *Potamogeton pectinatus* (**Table S1**), which was identified as a synonym of *Stuckenia pectinata*. Likewise, several genera within the Amaranthaceae family were formerly classified in the Chenopodiaceae (*Alternanthera*, *Atriplex*, *Axyris*, *Bassia*, *Suaeda* and *Salsola*) (**Table S1**), which could have influenced their classification. This suggests that there remains work to be done on the taxonomy of Amaranthaceae. There are also some genera in other families, such as *Cyperus* and *Panicum*, whose taxonomy is controversial (**Table S1**) and do not share the same photosynthetic pathway. Indeed, the photosynthetic pathway might be a good tool to explore species evolutionary history and taxonomy.

In all, our study, by identifying key patterns in the diversity and phylogenetic structure of C_4_ species across regions in China, provides an important advance in our understanding of their distributions. This provides basic information on consistent (across all taxa) and nuanced (different patterns within some families) patterns of diversity and phylogenetic clustering patterns in response to major climate gradients. Furthermore, understanding these patterns of distribution can provide an important foundation for the use of C_4_ species in restoration practices in degraded ecosystems.

## Data availability statement

The original contributions presented in the study are included in the article/**Supplementary Material**. Further inquiries can be directed to the corresponding author.

## Author contributions

AZ conceived the study. AZ, ZY, YZ, LM and HZ collected the data. AZ and LM analyzed the data. AZ wrote the manuscript. ZY, YZ, LM and HZ commented the manuscript. All authors gave final approval for publication. All authors contributed to the article.

## References

[B1] Edwards E. J. Still C. J. (2008). Climate, phylogeny and the ecological distribution of C_4_ grasses. Ecol. Lett. 11, 266–276. doi: 10.1111/J.1461-0248.2007.01144.X 18201200

[B2] Faith D. P. (1992). Conservation evaluation and phylogenetic diversity. Biol. Conserv. 61, 1–10. doi: 10.1016/0006-3207(92)91201-3

[B3] Jin Y. Qian H. (2022). V.PhyloMaker2: an updated and enlarged r package that can generate very large phylogenies for vascular plants. Plant Diversity 44 (4), 335–339. doi: 10.1016/j.pld.2022.05.005 35967255 PMC9363651

[B4] Kembel S. W. Cowan P. D. Helmus M. R. Cornwell W. K. Morlon H. Ackerly D. D.. (2010). Picante: r tools for integrating phylogenies and ecology. Bioinformatics 26 (11), 1463–1464. doi: 10.1093/bioinformatics/btq166 20395285

[B5] Latham R. E. Ricklefs R. E. (1993). Global patterns of tree species richness in moist forests: energy-diversity theory does not account for variation in species richness. Oikos 67, 325–333. doi: 10.2307/3545479

[B6] Liu H. Edwards E. J. Freckleton R. P. Osborne C. P. (2012). Phylogenetic niche conservatism in C_4_ grasses. Oecologia 170 (3), 835–845. doi: 10.1007/s00442-012-2337-5 22569558

[B7] Lu L. M. Mao L. F. Yang T. Ye J. F. Liu B. Li H. L.. (2018). Evolutionary history of the angiosperm flora of China. Nature 554 (7691), 234–238. doi: 10.1038/nature25485 29420476

[B8] Munroe S. E. McInerney F. A. Guerin G. R. Andrae J. W. Welti N. Caddy-Retalic S.. (2022). Plant families exhibit unique geographic trends in C4 richness and cover in Australia. PloS One 17 (8), e0271603. doi: 10.32942/osf.io/s4hb3 35994485 PMC9394836

[B9] Munson S. M. Long A. L. (2017). Climate drives shifts in grass reproductive phenology across the western USA. New Phytol. 213 (4), 1945–1955. doi: 10.1111/nph.14327 27870060

[B10] Osborne C. P. Salomaa A. Kluyver T. A. Visser V. Kellogg E. A. Morrone O.. (2015). A global database of C_4_ photosynthesis in grasses. New Phytol. 204, 441–446. doi: 10.1111/nph.12942 25046685

[B11] Pardo J. VanBuren R. (2021). Evolutionary innovations driving abiotic stress tolerance in C4 grasses and cereals. Plant Cell 33 (11), 3391–3401. doi: 10.1093/plcell/koab205 34387354 PMC8566246

[B12] Pyron R. A. Costa G. C. Patten M. A. Burbrink F. T. (2015). Phylogenetic niche conservatism and the evolutionary basis of ecological speciation. Biol. Rev. 90, 1248–1262. doi: 10.1111/brv.12154 25428167

[B13] Qian H. Deng T. Jin Y. Mao L. Ricklefs R. E. (2019). Phylogenetic dispersion and diversity in regional assemblages of seed plants in China. P. Natl. Acad. Sci. U.S.A. 116 (46), 23192–23201. doi: 10.1073/pnas.1822153116 PMC6859352 31659037

[B14] Qian H. Qian S. Sandel B. (2022). Phylogenetic structure of alien and native species in regional plant assemblages across China: testing niche conservatism hypothesis versus niche convergence hypothesis. Global Ecol. Biogeogr. 31 (9), 1864–1876. doi: 10.1111/geb.13566

[B15] Qian H. Sandel B. (2017). Phylogenetic structure of regional angiosperm assemblages across latitudinal and climatic gradients in north America. Global Ecol. Biogeogr. 26, 1258–1269. doi: 10.1111/geb.12634

[B16] R Core Team (2022). R: a language and environment for statistical computing (Vienna, Austria: R Foundation for Statistical Computing). Available at: https://www.R-project.org/.

[B17] Rudov A. Mashkour M. Djamali M. Akhani H. (2020). A review of C_4_ plants in southwest Asia: an ecological, geographical and taxonomical analysis of a region with high diversity of C_4_ eudicots. Front. Plant Sci. 11. doi: 10.3389/fpls.2020.546518 PMC7694577 33304357

[B18] Sage R. F. (2017). A portrait of the C_4_ photosynthetic family on the 50th anniversary of its discovery: species number, evolutionary lineages, and hall of fame. J. Exp. Bot. 68 (2), e11–e28. doi: 10.1093/jxb/erx005 28110278

[B19] Sage R. F. Monson R. K. (1999). C4 plant biology (New York, NY, USA: Academic Press). doi: 10.1016/B978-0-12-614440-6.X5000-9

[B20] Sieben E. J. J. Morris C. D. Kotze D. C. Muasya A. M. (2010). Changes in plant form and function across altitudinal and wetness gradients in the wetlands of the maloti-Drakensberg, south Africa. Plant Ecol. 207 (1), 107–119. doi: 10.1007/s11258-009-9657-5

[B21] Sonawane B. V. Koteyeva N. K. Johnson D. M. Cousins A. B. (2021). Differences in leaf anatomy determines temperature response of leaf hydraulic and mesophyll CO_2_ conductance in phylogenetically related C_4_ and C_3_ grass species. New Phytol. 230 (5), 1802–1814. doi: 10.1111/nph.17287 33605441

[B22] Taylor S. H. Hulme S. P. Rees M. Ripley B. S. Ian Woodward F. Osborne C. P. (2010). Ecophysiological traits in C_3_ and C_4_ grasses: a phylogenetically controlled screening experiment. New Phytol. 185 (3), 780–791. doi: 10.1111/j.1469-8137.2009.03102.x 20002318

[B23] The Angiosperm Phylogeny Group (2016). An update of the angiosperm phylogeny group classification for the orders and families of flowering plants: APG IV. bot. J. Linn. Soc 181 (1), 1–20. doi: 10.1111/boj.12385

[B24] Visser V. Woodward F. I. Freckleton R. P. Osborne C. P. (2012). Environmental factors determining the phylogenetic structure of C_4_ grass communities. J. Biogeogr. 39 (2), 232–246. doi: 10.1111/j.1365-2699.2011.02602.x

[B25] Wang R. Z. (2002a). Photosynthetic pathway types of forage species along grazing gradient from the songnen grassland, northeastern China. Photosynthetica 40, 57–61. doi: 10.1023/A:1020185906183

[B26] Wang R. Z. (2002b). Photosynthetic pathways and life forms in different grassland types from north China. Photosynthetica 40, 243–250. doi: 10.1023/A:1021301909227

[B27] Wang Z. Fang J. Tang Z. Lin X. (2011). Patterns, determinants and models of woody plant diversity in China. P. R. Soc B-Biol. Sci. 278 (1715), 2122–2132. doi: 10.1098/rspb.2010.1897 PMC3107620 21147804

[B28] Wang L. Jia Y. Zhang X. Qin H. (2015). Overview of higher plant diversity in China. Biodivers. Sci. 23 (2), 217–224. doi: 10.17520/biods.2015049

[B29] Wang R. Ma L. (2016). Climate-driven C4 plant distributions in China: divergence in C_4_ taxa. Sci. Rep-UK 6 (1), 1–8. doi: 10.1038/srep27977 PMC4908390 27302686

[B30] Webb C. O. Ackerly D. D. McPeek M. A. Donoghue M. J. (2002). Phylogenies and community ecology. Annu. Rev. Ecol. Evol. S. 33, 475–505. doi: 10.1146/annurev.ecolsys.33.010802.150448

[B31] Wiens J. J. Donoghue M. J. (2004). Historical biogeography, ecology, and species richness. Trends Ecol. Evol. 19, 639–644. doi: 10.1016/j.tree.2004.09.011 16701326

[B32] Zhang A. Xie Z. (2021). C_4_ herbs dominate the reservoir flood area of the three gorges reservoir. Sci. Total Environ. 755, 142479. doi: 10.1016/j.scitotenv.2020.142479 33035969

