# Supplementary material for: Geographic distribution of C4 species and its phylogenetic structure across China
Source: Front Plant Sci. 2023 Jun 8;14:1214980. doi: 10.3389/fpls.2023.1214980 (PMC10285315; doi:10.3389/fpls.2023.1214980)
Supplement: Supplementary file 1 [file DataSheet_1.docx]

**Table S2** Coefficient of Variation (CV) of species richness and three phylogenetic indices (PD, MPD and MNTD) at province level. CV was calculated as: Mean/ Standard Deviation.

| Assemblages | Indices | Mean | Standard Deviation | CV (%) |
| --- | --- | --- | --- | --- |
| All | Species richness | 212.4 | 71.3 | 33.5 |
| Poaceae | Species richness | 126.8 | 56.1 | 44.2 |
| Amaranthaceae | Species richness | 24.0 | 17.2 | 71.4 |
| Cyperaceae | Species richness | 34.8 | 18.6 | 53.4 |
| All | PD | 3148.6 | 620.6 | 19.7 |
| Poaceae | PD | 1045.4 | 389.1 | 37.2 |
| Amaranthaceae | PD | 284.3 | 157.4 | 55.4 |
| Cyperaceae | PD | 230.2 | 110.8 | 48.1 |
| All | MPD | 163.1 | 21.0 | 12.9 |
| Poaceae | MPD | 44.4 | 2.0 | 4.6 |
| Amaranthaceae | MPD | 55.1 | 10.9 | 19.9 |
| Cyperaceae | MPD | 29.8 | 5.4 | 17.9 |
| All | MNTD | 17.8 | 2.1 | 11.9 |
| Poaceae | MNTD | 12.1 | 1.7 | 13.9 |
| Amaranthaceae | MNTD | 13.4 | 3.2 | 23.5 |
| Cyperaceae | MNTD | 11.5 | 8.6 | 74.3 |
| All | SES.PD | -2.1 | 1.1 | -54.9 |
| Poaceae | SES.PD | -2.2 | 1.0 | -45.5 |
| Amaranthaceae | SES.PD | -2.4 | 0.7 | -27.0 |
| Cyperaceae | SES.PD | -1.1 | 0.9 | -87.9 |
| All | SES.MPD | -2.4 | 4.1 | -171.1 |
| Poaceae | SES.MPD | -1.3 | 1.5 | -115.9 |
| Amaranthaceae | SES.MPD | -0.8 | 1.6 | -186.7 |
| Cyperaceae | SES.MPD | -0.3 | 1.0 | -294.1 |
| All | SES.MNTD | -2.2 | 0.7 | -30.7 |
| Poaceae | SES.MNTD | -2.4 | 1.0 | -42.6 |
| Amaranthaceae | SES.MNTD | -2.0 | 0.6 | -28.1 |
| Cyperaceae | SES.MNTD | -1.7 | 1.2 | -70.0 |


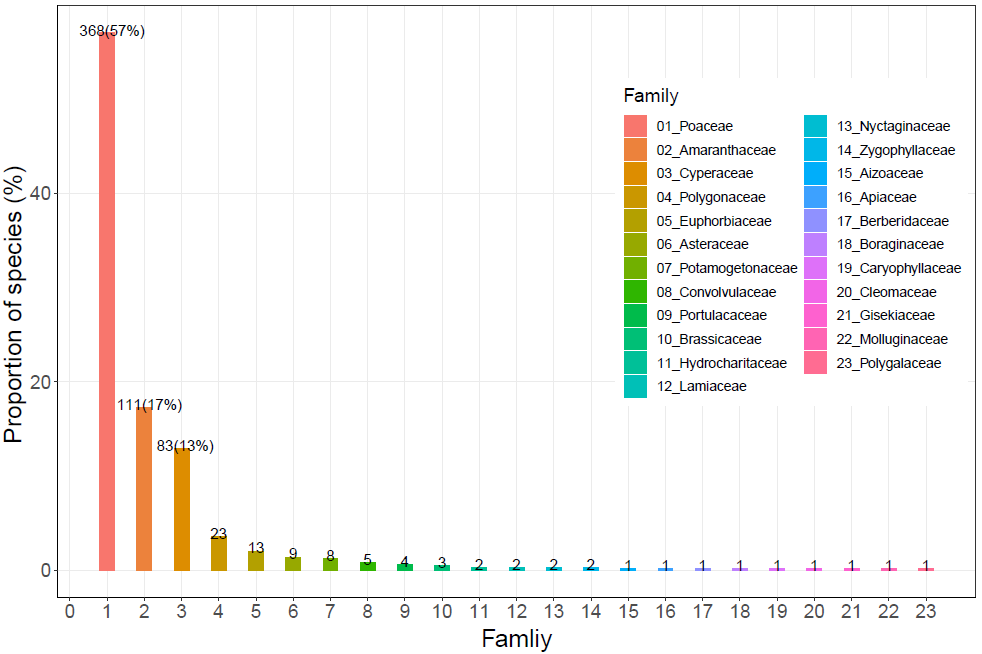


**Figure S1** Occupancy of different families of C_4_ plants distributed across China. The number on the top of each bar represents the number of species and its proportion of species.


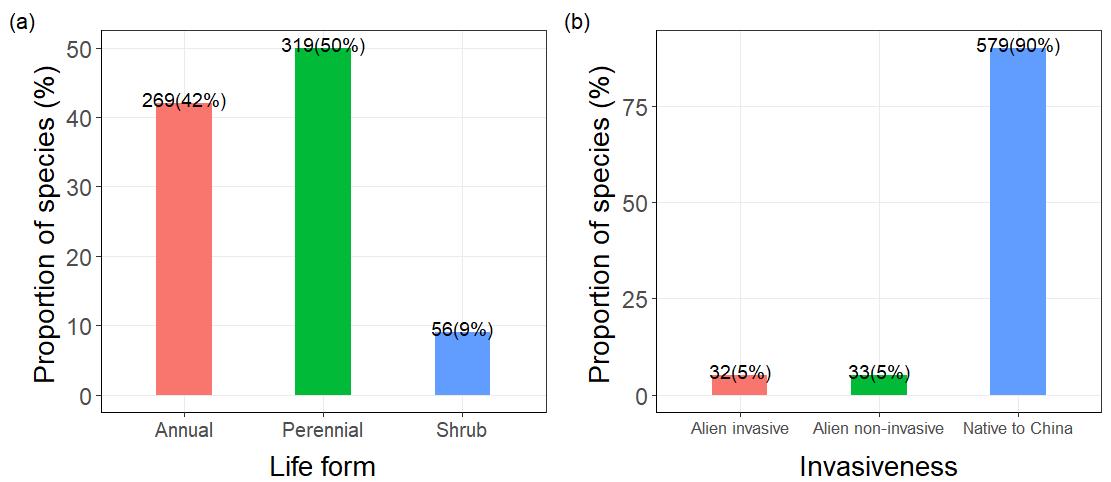


**Figure S2** Occupancy of different life forms (a) and invasiveness (b) of C_4_ plants distributed in China. The number on the top of each bar represents the number of species and its proportion of species.


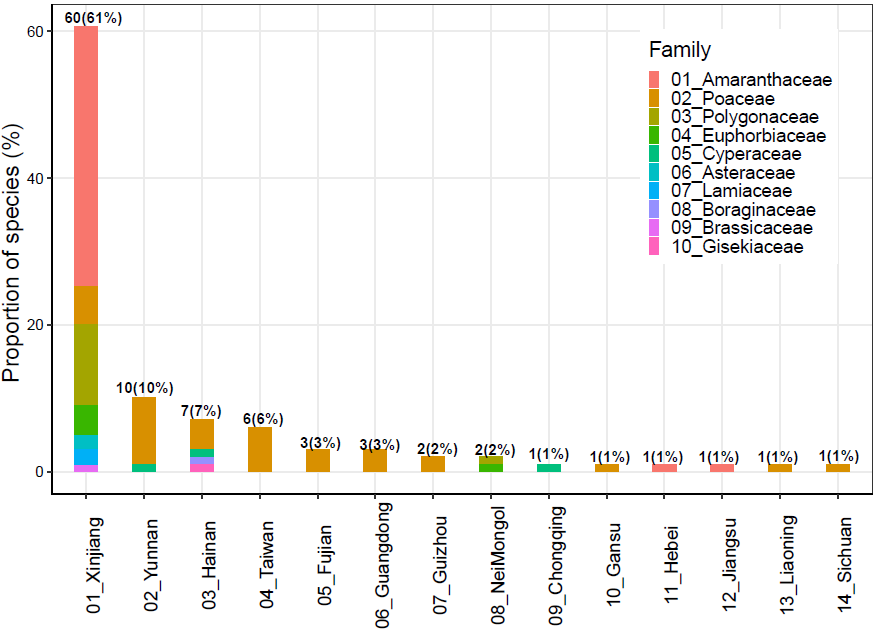


**Figure S3** Distribution of 99 C_4_ species that only occurred in one province. The number on the top of each bar represents the number of species and its proportion of species.


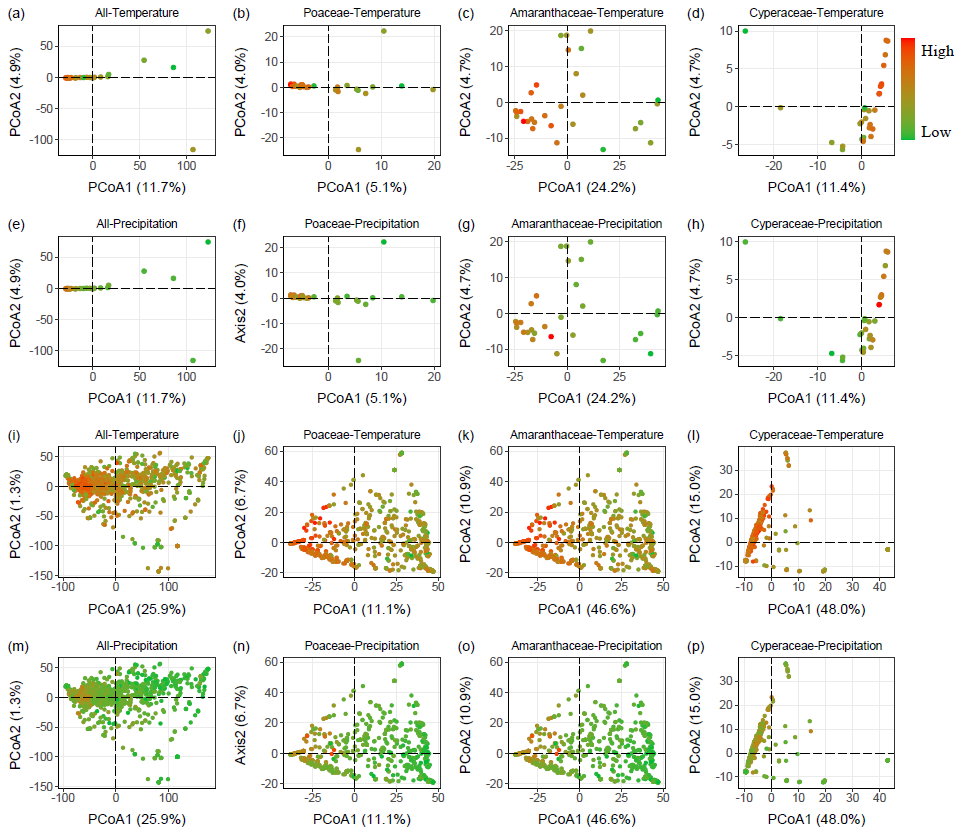


**Figure S4** Relative phylogenetic differences (MPD) among the regions (29 province level (a-h) and grid level (i-p)), visualized using a Principal Coordinates Analysis (PCoA) ordination biplot, calculated using the Gower distance metric. The color of dots varied from green to red, meaning the Temperature/Precipitation increased from low to high.


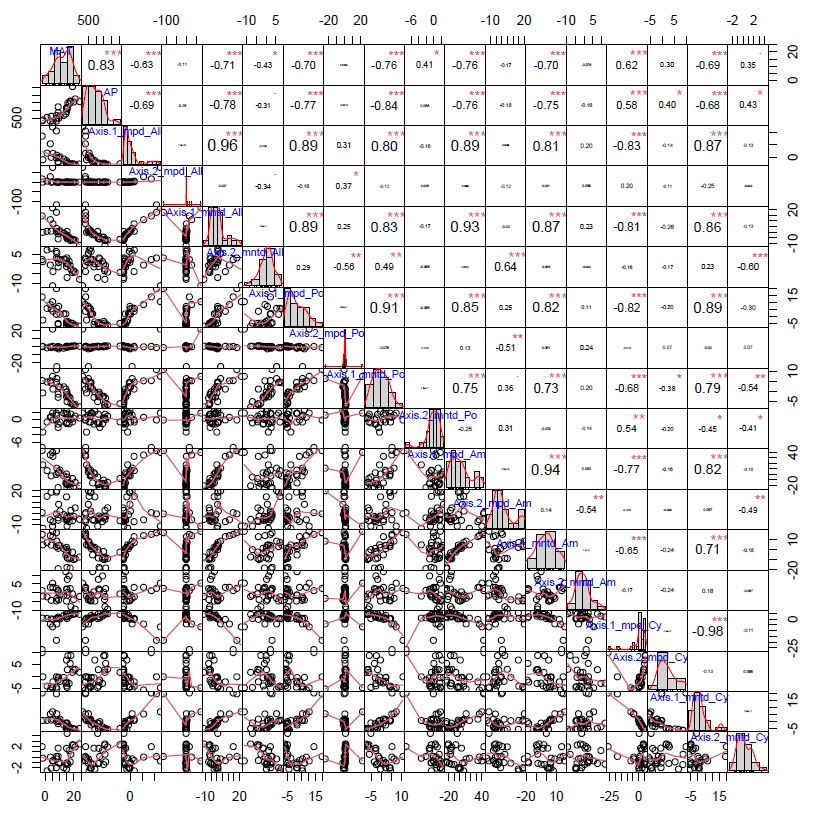


**Figure S5** Spearman's rank correlation of PCoA’s axes (Axis.1 and Axis.2) and phylogenetic difference (sesmpd, sesmntd) with mean annual temperature (MAT) and annual precipitation (AP) at regions of 29 province level, calculated by the R package *PerformanceAnalytics* (Peterson & Carl 2020). These correlations were calculated on four assemblages: All C_4_ species (All), Poaceae C_4_ species (Po), Amaranthaceae C_4_ species (Am) and Cyperaceae C_4_ species (Cy). *** indicates a significance at *P* < 0.001, ** indicates a significance at 0.001 < *P* < 0.01, * indicates a significance at 0.01 < *P* < 0.05, and no asterisk indicates non-significance at *P* > 0.05.


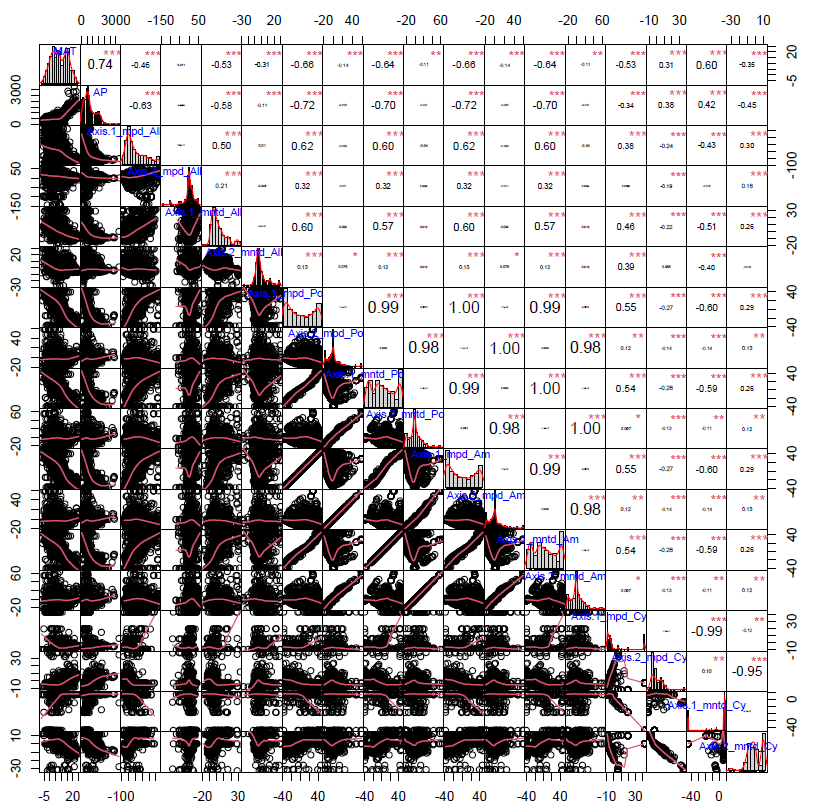


**Figure S6** Spearman's rank correlation of PCoA’s axes (Axis.1 and Axis.2) and phylogenetic difference (sesmpd, sesmntd) with mean annual temperature (MAT) and annual precipitation (AP) at regions of grid level (100km × 100km) , calculated by the R package *PerformanceAnalytics* (Peterson & Carl 2020). These correlations were calculated on four assemblages: All C_4_ species (All), Poaceae C_4_ species (Po), Amaranthaceae C_4_ species (Am) and Cyperaceae C_4_ species (Cy). *** indicates a significance at *P* < 0.001, ** indicates a significance at 0.001 < *P* < 0.01, * indicates a significance at 0.01 < *P* < 0.05, and no asterisk indicates non-significance at *P* > 0.05.

**References**

Peterson, B. G., Carl, P. (2020). PerformanceAnalytics: Econometric Tools for Performance and Risk Analysis. R package version 2.0.4, <https://CRAN.R-project.org/package=PerformanceAnalytics>.
